# Change in Urgency Status Among Ulcerative Colitis Patients: Understanding a Potential Unmet Patient Need From the CorEvitas Inflammatory Bowel Disease Registry

**DOI:** 10.1016/j.gastha.2023.03.024

**Published:** 2023-04-06

**Authors:** Douglas C. Wolf, April N. Naegeli, Page C. Moore, Jud C. Janak, Margaux M. Crabtree, Mingyang Shan, Theresa M. Hunter, Angelina Sontag, Raymond K. Cross

**Affiliations:** 1 Atlanta Gastroenterology Associates, Atlanta, Georgia; 2 Eli Lilly and Company, Indianapolis, Indiana; 3 CorEvitas LLC, Waltham, Massachusetts; 4 University of Maryland School of Medicine, Baltimore, Maryland

**Keywords:** Ulcerative colitis, Real-world studies, Patient-reported outcomes, Fecal urgency

## Abstract

**Background and Aims:**

Fecal urgency is a common symptom of Ulcerative Colitis (UC). We explored the association between changes in fecal urgency for patient characteristics and evaluated the association between change in treatment and change in fecal urgency.

**Methods:**

The study cohort (n = 400) included UC patients in the CorEvitas Inflammatory Bowel Disease Registry between May 3, 2017 and September 1, 2020. Fecal urgency was defined using the Simple Clinical Colitis Activity Index. Urgency groups were formed by urgency at enrollment and 6-month follow-up visit: no persistent urgency at both visits (NPU); change from urgency to no urgency (UN); change from no urgency to urgency (NU); and persistent urgency at both visits (PU). Descriptive statistics were used to explore between urgency group differences at baseline and Kaplan-Meier curves to compare time to first treatment change.

**Results:**

Groups included NPU (n = 175), UN (n = 86), NU (n = 56), and PU (n = 83). At enrollment, we found differences between groups for increased depression, anxiety, prior infections, diabetes; also, greater fatigue, pain, work impairment, work hours affected, and daily activities impacted. Compared to NPU patients, UN, NU, and PU patients were more likely to change treatment between enrollment and 6-month follow-up visit, and a higher proportion of UN, NU, and PU patients on a biologic at enrollment changed treatment vs the NPU group between both visits.

**Conclusion:**

Among real-world UC patients, fecal urgency status is associated with increased comorbidities and worse patient-reported outcomes and significant differences in change of treatment and time to treatment change. Urgency at any time point diminishes quality of life and may be a sign of inadequate therapy, which often is an indication to switch therapy.

Ulcerative Colitis (UC) is an idiopathic chronic inflammatory condition of the rectum and colon. The disease presents with various degrees of clinical activity and severity and is associated with significant morbidity.^1^ Fecal urgency, also referred to as bowel movement urgency, is the sudden need for a bowel movement. Of the many symptoms associated with UC, fecal urgency is one of UC patients' most common and troublesome symptoms and affects patients with both active and inactive disease.^2^, ^3^, ^4^, ^5^ Farrell et al^3^ found that fecal urgency was prevalent in 84% of the patients with active disease and 50% of the patients with inactive disease in their study of 247 UC patients from a university hospital outpatient clinic.

Moreover, fecal urgency is a predictor consistently associated with fecal incontinence.^6^, ^7^, ^8^, ^9^, ^10^ The importance of fecal urgency is immeasurable as studies have consistently shown that UC patients with fecal urgency are more likely to experience comorbid psychological conditions (ie, anxiety and depression), weakened social interactions or career progression, and impaired quality of life.^11^, ^12^, ^13^, ^14^, ^15^ Additionally, in a recent cross-sectional study combined with a 12-month longitudinal cohort in a patient-powered research network, Sninsky et al found that urgency was associated with a diminished quality of life.^15^

Fecal urgency is a factor in the diagnosis and treatment of UC by the American College of Gastroenterology. The American College of Gastroenterology Clinical Guidelines (March 2019) recognize fecal urgency as a component of a UC evaluation. A primary goal of UC treatment is to restore normal bowel function by mitigating the underlying disease that leads to urgency and related symptoms.^16^ If treatment is not received or is ineffective, patients will continue to suffer from the impact of fecal urgency and the major symptoms associated with UC (ie, abdominal pain, rectal bleeding, and diarrhea).^1^ Furthermore, given the association between fecal urgency and decreased quality of life, routine assessment for the potential causes of this demoralizing symptom (ie, persistent inflammation, structural damage, anorectal dysfunction) is needed to improve outcomes.

To address fecal urgency in a real-world population, our exploratory study was developed using patients from one of the newest Inflammatory Bowel Disease (IBD) registries in the United States. Our cross-sectional analysis aimed to (1) examine the association between change in fecal urgency (from enrollment to the 6-month follow-up visit) and sociodemographics, lifestyle characteristics, history of comorbidities, disease characteristics, disease activity, past medication use, and patient-reported outcomes (PROs) and (2) evaluate the association between change in treatment and change in fecal urgency (from enrollment to the 6-month follow-up visit).

## Patients and methods

### Registry overview

Launched in May 2017, the CorEvitas (formerly known as Corrona) IBD Registry is a prospective, noninterventional, research study of patients with IBD aged at least 18 years or more under the care of a board-certified gastroenterologist. To better focus on the study of safety and effectiveness of biologic and small molecule therapies explicitly used in the treatment of UC and Crohn’s disease, in January 2019, the Registry changed the protocol only to enroll patients initiating biologic/Janus kinase inhibitor (JAKi) therapy. Newly enrolled patients were required to have initiated or switched to an approved biologic or JAKi to treat UC or Crohn’s disease within the previous 12 months. Eligible medications for enrollment include Food and Drug Administration–approved biologic treatments for IBD (eg, tumor necrosis factor inhibitors, an interleukin-12/23 inhibitor, an integrin α4β7 inhibitor, an integrin α4 inhibitor, and JAKi).

Enrollment data are collected from the patients and their treating gastroenterologists during routine clinical visits using CorEvitas Registry questionnaires. Data include patient demographics, lifestyle characteristics, history of comorbidities, disease characteristics, disease activity, past and current medication use, and PROs. As of September 30, 2020, the CorEvitas IBD Registry database included 2184 patients and 4765 patient visits, with a mean patient follow-up duration of 1.3 years (median 1.2 years). Patients were recruited from 67 private and academic practice sites across 21 states in the United States, with 144 participating gastroenterologists.

### Patient population

**Figure 1:**
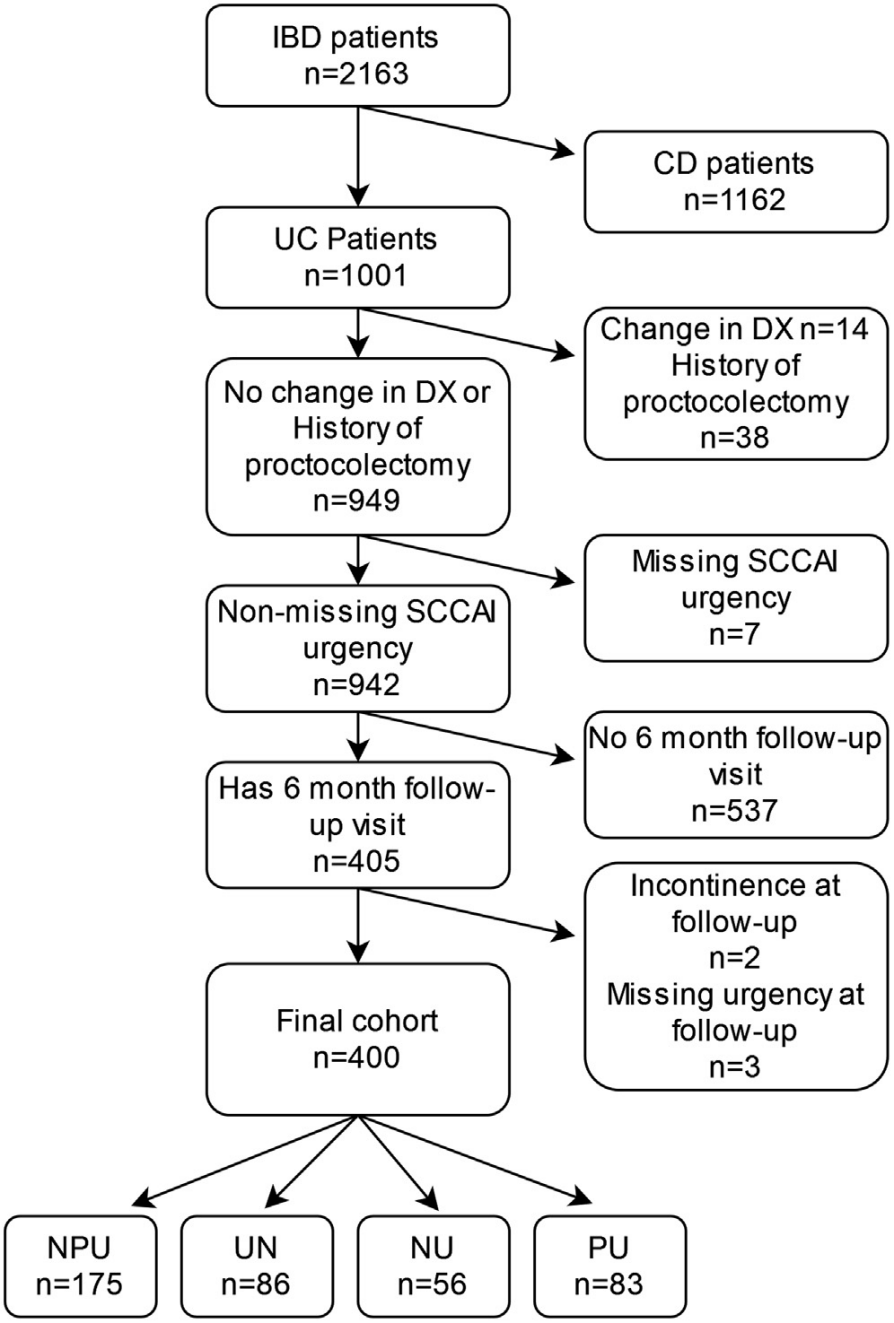
Patient attrition flow chart.

Our study cohort included UC patients (whose diagnosis has not changed at subsequent follow-up visits) enrolled in the CorEvitas IBD Registry from May 3, 2017 to September 1, 2020, with nonmissing fecal urgency data at enrollment and at the 6-month follow-up visit (Figure 1). UC patients with a history of proctocolectomy (yes or unknown), J-pouch, or history of pouchitis were excluded from this analysis.

Fecal urgency was defined using the Simple Clinical Colitis Activity Index (SCCAI); urgency of defecation item response options included none or hurry/immediate categories.^17^ The incontinence response option was excluded in the analysis due to the small number of patients with this response. Based on changes in fecal urgency from enrollment to the 6-month follow-up visit, the study cohort was stratified into 4 mutually exclusive groups: patients with no fecal urgency at enrollment or the 6-month follow-up visit (no persistent urgency [NPU]); patients who reported a resolution of fecal urgency after enrollment to the 6-month follow-up visit (urgency to none [UN]); patients who reported onset of fecal urgency after enrollment to the 6-month follow-up visit (none to urgency [NU]); and patients with fecal urgency at both enrollment and the 6-month follow-up visit (persistent urgency [PU]).

### Assessment of patient characteristics and outcomes

Sociodemographic characteristics collected at enrollment included age, gender, race (White or non-White), ethnicity (Hispanic or non-Hispanic), body mass index, type of health insurance plan, and education. History of physician-reported comorbidities included cardiovascular, autoimmune, gastrointestinal, respiratory, digestive/hepatic, neurologic diseases, cancer, infections, diabetes, osteoporosis, depression, anxiety, and other nonserious medical conditions. We used a modified version of the Charlson Comorbidity Index calculated as the sum of comorbid conditions available in the registry.^18^ Disease activity was measured using the Mayo Score (Partial) (Remission [0–1], mild disease [2–4], moderate disease [5–6], and severe disease [7–9]).^19^ Disease characteristics included location of disease (proctitis, left-sided, pan-colitis), history of hospitalization and emergency room use for UC-related issues, and difference in time between symptom onset and UC diagnosis (years). History of extraintestinal manifestations (eg, arthritis, skin manifestations, and eye involvement) was collected.

Data included the history of previous drug therapies comprised of the number of prior biologics and JAKi, prior 5-aminosalicylic acids (5-ASAs), prior immunosuppressant therapies, corticosteroid use, antibiotic use, and the duration of current UC therapy. Providers record the patient’s complete IBD treatment history, current medications, and any changes for drugs prescribed at enrollment and for drugs started and/or stopped less than 2 years prior to enrollment including dose and frequency information.

PROs to measure health status and function included the use of the Patient-Reported Outcomes Measurement Information System (PROMIS) questionnaires to measure fatigue, sleep disturbance, pain interference, depression, and anxiety. Higher scores indicate poorer health. PROMIS scores are grouped as within normal limits (< 55), mild (55 to < 60), moderate (60 to < 70), and severe (≥ 70) based on domain scores measured in the US general population.^20^^,^^21^

Outcomes from the Work Productivity and Activity Impairment (WPAI) questionnaire were evaluated. The WPAI measures absenteeism (work hours missed), presenteeism (impairment at work/reduced on-the-job effectiveness), work productivity loss (overall work impairment/absenteeism plus presenteeism) for currently employed patients, and activity impairment (daily activities impaired) for all patients. The WPAI outcomes are scored as percentages of impairment, with higher numbers indicating more significant impairment and less productivity (ie, worse outcomes).^22^

### Statistical analysis

Descriptive statistics from the patient’s enrollment visit were reported for sociodemographics, lifestyle characteristics, history of comorbidities, disease characteristics, disease severity, past medication usage, and PROs for each fecal urgency group. Continuous variables were summarized by the number of observations, mean, and standard deviation; categorical variables were summarized by frequencies and percentages. If present, missing data were indicated by reduced sample sizes for variables. To quantify between group differences, the mean or proportional differences and their corresponding 95% confidence intervals were calculated. To determine outcomes for the WPAI, differences in each WPAI domain were also evaluated by comparing the proportion of subjects experiencing no (0%) vs any (> 0%) impairment.

The purpose of this unadjusted cross-sectional analysis was to highlight any differences in outcome measures between patient groups and not a measure of a difference in response to treatment. Chi-square tests were used to explore the association between change in treatment and change in fecal urgency. Kaplan-Meier curves and log-rank tests were used to compare time to first treatment change between fecal urgency status groups. R version 3.6.2 (The R Foundation for Statistical Computing) was used for analyses.

## Results

### Comparisons between urgency groups at enrollment

Table 1 presents the characteristics of the 400 patients who participated in this study and were categorized into 4 mutually exclusive fecal urgency groups: (1) NPU, n = 175 (44%); (2) UN, n = 86 (21%); (3) NU, n = 56 (14%); and (4) PU, n = 83 (21%). A higher proportion of UN patients (59%) and PU patients (60%) were female compared to NPU patients (44%). More UN patients (72%) were married or partnered compared to NPU patients (54%). However, history of comorbidities was similar across all fecal urgency groups except for depression (UN [13%], NU [13%], PU [13%] vs NPU [5%]), anxiety (PU [24%] vs NPU [10%]), prior infections (PU [24%] vs NPU [13%]), and diabetes (UN [14%], NU [9%], PU [8%] vs NPU [5%]). Disease characteristics were similar at enrollment except for proctitis across all 4 urgency groups. Compared to NPU patients (9%), the prevalence of proctitis was roughly double for all other groups (UN [19%], NU [16%], and PU [19%]).

**Table 1:** Ulcerative Colitis (UC) Patient Sociodemographics, History of Comorbidities, Disease Characteristics, and Disease Severity at Enrollment Visit, Stratified by Change in Physician-Reported Fecal Urgency From Time of Enrollment Visit to 6-Mo Follow-Up Visit

	No persistent Urgency (NPU)^f^ N = 175	Urgency to none (UN)^f^< N = 86	None to urgency (NU)^f^< N = 56	Persistent Urgency (PU)^f^ N = 83	Difference (95% CI)^g^ UN vs NPU<	Difference (95% CI)^g^ NU vs NPU<	Difference (95% CI)^g^ PU vs NPU<
Demographics and lifestyle characteristics							
Age in (y), mean (SD)	49.5 (17.4)	46.8 (15.6)	45.7 (16.9)	46.0 (14.6)	−2.7 (−6.9, 1.5)	−3.8 (−9.0, 1.4)	−3.5 (−7.5, 0.6)
Gender-female, n (%)	77/175 (44.0%)	50/86 (58.8%)	28/56 (50.0%)	50/83 (60.2%)	14.8 (2.0, 27.6)	6.0 (−9.0, 21.0)	16.2 (3.4, 29.1)
Race-White, n (%)	149/175 (85.1%)	76/86 (88.4%)	50/56 (89.3%)	72/83 (86.7%)	3.2 (−5.4, 11.8)	4.1 (−5.5, 13.8)	1.6 (−7.4, 10.6)
Marital status-Married/Partnered, n (%)	94/175 (53.7%)	61/86 (71.8%)	29/56 (51.8%)	46 (55.4%)	18.0 (6.0, 30.1)	−1.9 (−16.9, 13.1)	1.7 (−11.3, 14.7)
BMI (kg/m^2^)^a^, mean (SD)	27.6 (5.8)	29.0 (7.7)	28.9 (6.3)	27.6 (6.9)	1.4 (−0.5, 3.2)	1.3 (−0.6, 3.2)	−0.1 (−1.8, 1.7)
History of comorbidities and infections							
CVD^b^, n (%)	52/175 (29.7%)	27/86 (31.4%)	12/56 (21.4%)	27/83 (32.5%)	1.7 (−10.2, 13.6)	−8.3 (−21.0, 4.4)	2.8 (−9.3, 15.0)
Cancer^c^, n (%)	14/175 (8.0%)	5/86 (5.8%)	2/56 (3.6%)	7/83 (8.4%)	−2.2 (−8.6, 4.2)	−4.4 (−10.7, 1.9)	0.4 (−6.8, 7.6)
Diabetes, n (%)	9/175 (5.1%)	12/86 (14.0%)	5/56 (8.9%)	7/83 (8.4%)	8.8 (0.8, 16.8)	3.8 (−4.4, 11.9)	3.3 (−3.5, 10.1)
Depression, n (%)	8/175 (4.6%)	11/86 (12.8%)	7/56 (12.5%)	11/83 (13.3%)	8.2 (0.5, 15.9)	7.9 (−1.3, 17.1)	8.7 (0.8, 16.6)
Anxiety, n (%)	18/175 (10.3%)	11/86 (12.8%)	5/56 (8.9%)	20/83 (24.1%)	2.5 (−5.9, 10.9)	−1.4 (−10.1, 7.4)	13.8 (3.6, 24.1)
Infections^d^, n (%)	23/175 (13.1%)	9/86 (10.5%)	6/56 (10.7%)	20/83 (24.1%)	−2.7 (−10.9, 5.5)	−2.4 (−12.0, 7.1)	11.0 (0.5, 21.4)
C. Difficile colitis, n (%)	8/175 (4.6%)	3/86 (3.5%)	0/56 (0.0%)	7/83 (8.4%)	−1.1 (−6.0, 3.9)	−4.6 (−7.7, −1.5)	3.9 (−2.9, 10.6)
Disease characteristics							
Proctitis, n (%)	16/175 (9.1%)	16/86 (18.6%)	9/56 (16.1%)	16/83 (19.3%)	9.5 (0.2, 18.7)	6.9 (−3.6, 17.5)	10.1 (0.6, 19.6)
Left side disease, n (%)	81/175 (46.3%)	37/86 (43.0%)	26/56 (46.4%)	38/83 (45.8%)	−3.3 (−16.1, 9.5)	0.1 (−14.9, 15.1)	−0.5 (−13.5, 12.5)
Pan-colitis, n (%)	78/175 (44.6%)	34/86 (39.5%)	23/56 (41.1%)	33/83 (39.8%)	−5.0 (−17.7, 7.6)	−3.5 (−18.3, 11.3)	−4.8 (−17.7, 8.0)
Time since UC symptom onset (y)^e^, mean (SD)	11.6 (10.4)	11.0 (10.8)	10.2 (9.0)	11.3 (9.5)	−0.6 (−3.4, 2.2)	−1.4 (−4.2, 1.5)	−0.3 (−2.8, 2.3)
Time since UC diagnosis (y), mean (SD)	10.6 (9.9)	10.3 (10.7)	9.1 (7.9)	10.3 (8.8)	−0.3 (−3, 2.4)	−1.5 (−4.1, 1.0)	−0.4 (−2.8, 2.0)
Disease severity							
Mayo score (partial) (0–9)							
Remission (0–1), n (%)	120/169 (71.0%)	36/85 (42.4%)	37/54 (68.5%)	18/77 (23.4%)	−28.7 (−41.2, −16.1)	−2.5 (−16.6, 11.7)	−47.6 (−59.3, −36)
Mild disease (2–4), n (%)	46/169 (27.2%)	31/85 (36.5%)	13/54 (24.1%)	39/77 (50.6%)	9.2 (−3.0, 21.5)	−3.1 (−16.4, 10.1)	23.4 (10.4, 36.5)
Moderate/Severe disease (5–9), n (%)	3/169 (1.8%)	18/85 (21.2%)	4/54 (7.4%)	20/77 (26.0%)	19.4 (10.5, 28.3)	5.6 (−1.6, 12.9)	24.2 (14.2, 34.2)
≥ 1 stool per day more than normal, n (%)	40/167 (23.7%)	44/84 (51.8%)	14/52 (25.9%)	52/75 (67.5%)	28.1 (15.7, 40.5)	2.3 (−11.1, 15.6)	43.9 (31.6, 56.1)
Rectal bleeding, blood seen, n (%)	16/169 (9.5%)	33/85 (38.8%)	8/54 (14.8%)	33/77 (42.9%)	29.3 (18.1, 40.6)	5.3 (−5.1, 15.8)	33.4 (21.5, 45.3)
Simple clinical colitis activity index (SCCAI)							

SCCAI, mean (SD)	0.6 (1.1)	3.6 (2.2)	0.9 (1.2)	4.5 (2.4)	2.9 (2.4, 3.5)	0.3 (−0.1, 0.7)	3.9 (3.3, 4.5)
SCCAI (< 2.5) remission, n (%)	153/175 (87.4%)	37/86 (43.0%)	46/56 (82.1%)	14/83 (16.9%)	−44.4 (−56, −32.9)	−5.3 (−16.5, 5.9)	−70.6 (−80.0, −61.1)
Bowel frequency							
Bowel frequency (d), 0–3, n (%)	165/175 (94.3%)	56/86 (65.1%)	50/56 (89.3%)	41/83 (49.4%)	−29.2 (−39.8, −18.5)	−5.0 (−13.8, 3.8)	−44.9 (−56.2, −33.6)
Bowel frequency (d), ≥ 4, n (%)	10/175 (5.7%)	30/86 (34.9%)	6/56 (10.7%)	42/83 (50.6%)	29.2 (18.5, 39.8)	5.0 (−3.8, 13.8)	44.9 (33.6, 56.2)
Bowel frequency (night), 0, n (%)	151/175 (86.3%)	47/86 (54.7%)	50/56 (89.3%)	33/81 (40.7%)	−31.6 (−43.3, −19.9)	3.0 (−6.6, 12.6)	−45.6 (−57.4, −33.7)
Bowel frequency (night), ≥ 1, n (%)	24/175 (13.7%)	39/86 (45.3%)	6/56 (10.7%)	48/81 (59.3%)	31.6 (19.9, 43.3)	−3.0 (−12.6, 6.6)	45.6 (33.7, 57.4)

SD, standard deviation; BMI, body mass index; CVD, cardiovascular disease.

a BMI, NPU, n = 174; UN, n = 86; NU, n = 56; PU, n = 83.

b CVD, includes cardiac revascularization procedure, ventricular arrhythmia, cardiac arrest, myocardial infarction, acute coronary syndrome, unstable angina, coronary artery disease, congestive heart failure, and cerebrovascular disease (stroke, TIA, peripheral vascular disease, peripheral arterial disease).

c Colonic dysplasia, colon cancer, lymphoma, lung cancer, breast cancer, skin cancer (basal cell), skin cancer (melanoma), premalignancy, and other cancer.

d Bronchitis, *C difficile* colitis, candida, diverticulitis, gastroenteritis, herpes zoster, joint/bursa, meningitis/encephalitis, pneumonia, sepsis, sinusitis, upper respiratory infection (URI), urinary tract infection (UTI), TB, and other infection.

e Time since UC, symptom onset, NPU, n = 172; UN, n = 85; NU, n = 56; PU, n = 82.

f NPU, no persistent urgency at enrollment or follow-up; UN, reported resolution of urgency; NU, reported onset of urgency after enrollment to follow-up after enrollment to follow-up; PU, reported urgency at both enrollment and follow-up.

g Unadjusted mean difference (95% CI) between groups for continuous variables and difference in percentage points (95% CI) between groups for categorical variables.

As presented in Table 2, compared to NPU patients, a higher proportion of NU patients experienced worse PROs and had a greater proportion of moderate-to-severe PROMIS fatigue (25% vs 11%), depression (14% vs 5%), and anxiety (25% vs 9%) at enrollment. Furthermore, a markedly lower percentage of NPU patients suffered UC-related work impairment (37%), work hours affected (38%), and daily activities impacted (40%) vs UN patients (67%, 68%, 75%), NU patients (58%, 58%, 59%), and PU patients (91%, 92%, 93%), respectively.

**Table 2:** Ulcerative Colitis (UC) Past Medications and Patient-Reported Outcomes (PROs) at Enrollment Visit, Stratified by Change in Physician-Reported Fecal Urgency From Time of Enrollment Visit to 6-Mo Follow-Up Visit

	No persistent Urgency (NPU)^a^ N = 175	Urgency to none (UN)^a^< N = 86	None to urgency (NU)^a^< N = 56	Persistent Urgency (PU)^a^ N = 83	Difference (95% CI)^b^ UN vs NPU<	Difference (95% CI)^b^ NU vs NPU<	Difference (95% CI)^b^ PU vs NPU<
Past medications							
Biologic experienced, n (%)	30/175 (17.1%)	18/86 (20.9%)	10/56 (17.9%)	21/83 (25.3%)	3.8 (−6.5, 14.0)	0.7 (−10.8, 12.2)	8.2 (−2.7, 19.1)
Number of prior biologics/JAKi							
0	145/175 (82.9%)	68/86 (79.1%)	46/56 (82.1%)	62/83 (74.7%)	−3.8 (−14.0, 6.5)	−0.7 (−12.2, 10.8)	−8.2 (−19.1, 2.7)
1	19/175 (10.9%)	15/86 (17.4%)	5/56 (8.9%)	14/83 (16.9%)	6.6 (−2.7, 15.8)	−1.9 (−10.7, 6.8)	6.0 (−3.3, 15.3)
2+	11/175 (6.3%)	3/86 (3.5%)	5/56 (8.9%)	7/83 (8.4%)	−2.8 (−8.1, 2.5)	2.6 (−5.7, 10.9)	2.1 (−4.8, 9.1)
TNFi experienced, n (%)	153/175 (87.4%)	71/86 (82.6%)	51/56 (91.1%)	66/83 (79.5%)	−4.9 (−14.3, 4.5)	3.6 (−5.3, 12.6)	−7.9 (−17.9, 2.1)
Integrin α4 β7 experienced, n (%)	31/175 (17.7%)	14/86 (16.3%)	12/56 (21.4%)	18/83 (21.7%)	−1.4 (−11.1, 8.2)	3.7 (−8.4, 15.9)	4 (−6.5, 14.5)
IST experienced, n (%)	51/175 (29.1%)	17/86 (19.8%)	12/56 (21.4%)	24/83 (28.9%)	−9.4 (−20.1, 1.4)	−7.7 (−20.4, 5.0)	−0.2 (−12.1, 11.6)
5-ASA experienced, n (%)	143/175 (81.7%)	71/86 (82.6%)	48/56 (85.7%)	67/83 (80.7%)	0.9 (−9.0, 10.7)	4.0 (−6.8, 14.8)	−1.0 (−11.2, 9.2)
Corticosteroid experienced, n (%)	104/175 (59.4%)	55/86 (64.0%)	40/56 (71.4%)	55/83 (66.3%)	4.5 (−8.0, 17.0)	12.0 (−1.9, 25.9)	6.8 (−5.7, 19.3)
Antibiotic experienced, n (%)	24/175 (13.7%)	23/86 (26.7%)	8/56 (14.3%)	15/83 (18.1%)	13.0 (2.4, 23.7)	0.6 (−9.9, 11.1)	4.4 (−5.4, 14.1)
Patient-reported outcomes (PROs)							
Patient-reported outcomes measurement information system (PROMIS)							
Fatigue, mean (SD)	46.1 (10.1)	52.9 (9.9)	51.3 (9.8)	55.4 (9.4)	6.9 (4.2, 9.5)	5.2 (2.2, 8.2)	9.3 (6.8, 11.9)
Fatigue–Categorical							
Within normal limits (< 55)	140/172 (81.4%)	41/84 (48.8%)	32/56 (57.1%)	39/82 (47.6%)	−32.6 (−44.8, −20.4)	−24.3 (−38.5, −10.1)	−33.8 (−46.1, −21.6)
Mild (55–60)	14/172 (8.1%)	20/84 (23.8%)	10/56 (17.9%)	14/83 (17.1%)	15.7 (5.7, 25.7)	9.7 (−1.1, 20.6)	8.9 (−0.2, 18.0)
Moderate/Severe (≥ 60)	18/172 (10.5%)	23/84 (27.4%)	14/56 (25.0%)	29/83 (35.4%)	16.9 (6.3, 27.5)	14.5 (2.3, 26.8)	24.9 (13.6, 36.2)
Sleep disturbance, mean (SD)	47.9 (7.6)	50.7 (8.0)	49.5 (8.9)	55.0 (7.7)	2.8 (0.8, 4.9)	1.6 (−1.1, 4.2)	7.1 (5.1, 9.1)
Sleep disturbance–Categorical							
Within normal limits (< 55)	153/174 (87.9%)	62/85 (72.9%)	42/56 (75.0%)	48/83 (57.8%)	−15 (−25.6, −4.4)	−12.9 (−25.3, −0.6)	−30.1 (−41.8, −18.4)
Mild (55–60)	16/174 (9.2%)	14/85 (16.5%)	10/56 (17.9%)	15/83 (18.1%)	7.3 (−1.7, 16.2)	8.7 (−2.3, 19.6)	8.9 (−0.5, 18.2)
Moderate/Severe (≥ 60)	5/174 (2.9%)	9/85 (10.6%)	4/56 (7.1%)	20/83 (24.1%)	7.7 (0.7, 14.7)	4.3 (−2.9, 11.5)	21.2 (11.7, 30.8)
Pain interference, mean (SD)	46.1 (7.4)	52.3 (9.6)	49.1 (8.3)	55.1 (9.4)	6.2 (3.8, 8.5)	3.0 (0.5, 5.5)	9.0 (6.7, 11.3)
Pain interference–Categorical							
Within normal limits (< 55)	143/175 (81.7%)	42/85 (49.4%)	37/56 (66.1%)	32/82 (39.0%)	−32.3 (−44.4, −20.2)	−15.6 (−29.3, −2)	−42.7 (−54.7, −30.7)
Mild (55–60)	22/175 (12.6%)	17/85 (20.0%)	10/56 (17.9%)	19/82 (23.2%)	7.4 (−2.4, 17.3)	5.3 (−5.9, 16.5)	10.6 (0.2, 21.0)
Moderate/Severe (≥ 60)	10 (5.7%)	26 (30.6%)	9 (16.1%)	31 (37.8%)	24.9 (14.5, 35.3)	10.4 (0.1, 20.6)	32.1 (21.0, 43.1)
Depression, mean (SD)	44.7 (6.9)	45.8 (7.0)	48.2 (9.5)	50.2 (9.0)	8.2 (0.5, 15.9)	7.9 (−1.3, 17.1)	8.7 (0.8, 16.6)
Depression–Categorical							
Within normal limits (< 55)	151/175 (86.3%)	70/85 (82.4%)	39/56 (69.6%)	53/83 (63.9%)	−3.9 (−13.5, 5.6)	−16.7 (−29.7, −3.6)	−22.4 (−34, −10.9)
Mild (55–60)	16/175 (9.1%)	10/86 (11.8%)	9/56 (16.1%)	16/83 (19.3%)	2.6 (−5.5, 10.7)	6.9 (−3.6, 17.5)	10.1 (0.6, 19.6)
Moderate/Severe (≥ 60)	8/175 (4.6%)	5/85 (5.9%)	8/56 (14.3%)	14/83 (16.9%)	1.3 (−4.6, 7.2)	9.7 (0.0, 19.4)	12.3 (3.7, 20.9)
Anxiety, mean (SD)	47.6 (8.6)	50.9 (9.4)	50.1 (10.5)	55.2 (8.8)	2.5 (−5.9, 10.9)	−1.4 (−10.1, 7.4)	13.8 (3.6, 24.1)
Anxiety–categorical							
Within normal limits (< 55)	132/174 (75.9%)	50/85 (58.8%)	34/56 (60.7%)	36/83 (43.4%)	−17.0 (−29.3, −4.8)	−15.1 (−29.4, −0.9)	−32.5 (−44.9, −20.1)
Mild (55–60)	26/174 (14.9%)	17/85 (20.0%)	8/56 (14.3%)	22/83 (26.5%)	5.1 (−5.0, 15.1)	−0.7 (−11.2, 9.9)	11.6 (0.7, 22.4)
Moderate/Severe (≥ 60)	16/174 (9.2%)	18/85 (21.2%)	14/56 (25.0%)	25/83 (30.1%)	12.0 (2.3, 21.7)	15.8 (3.7, 27.9)	20.9 (10.2, 31.7)
Work productivity activity impairment (WPAI)							
Current employment, n (%)	122/175 (69.7%)	58/85 (68.2%)	33/56 (58.9%)	55/83 (66.3%)	−1.5 (−13.5, 10.5)	−10.8 (−25.4, 3.8)	−3.4 (−15.7, 8.8)
Work hours missed due to UC, n (%)	6/109 (5.5%)	14/57 (24.6%)	6/33 (18.2%)	17/52 (32.7%)	19.1 (7.1, 31.0)	12.7 (−1.2, 26.5)	27.2 (13.7, 40.6)
Impairment while working due to UC, n (%)	45/122 (36.9%)	39/58 (67.2%)	19/33 (57.6%)	49/54 (90.7%)	30.3 (15.5, 45.2)	20.7 (1.8, 39.6)	53.8 (42.3, 65.4)
Work hours affected by UC, n (%)	41/109 (37.6%)	39/57 (68.4%)	19/33 (57.6%)	47/51 (92.2%)	30.8 (15.7, 45.9)	20.0 (0.8, 39.1)	54.5 (42.8, 66.3)
Daily activities affected by UC, n (%)	70/174 (40.2%)	64/85 (75.3%)	33/56 (58.9%)	77/83 (92.8%)	35.1 (23.3, 46.8)	18.7 (3.9, 33.5)	52.5 (43.4, 61.7)

aSCCAI, NPU, n = 163; UN, n = 82; NU, n = 53; PU, n = 83.

JAKi, Janus kinase inhibitors; TNFI, tumor necrosis factor inhibitors; 5-ASAs, 5-aminosalicylic acids; ISTs, immunosuppressant therapies; SD, standard deviation.

a NPU, no persistent urgency at enrollment or follow-up; UN, reported resolution of urgency after enrollment to follow-up; NU, reported onset of urgency after enrollment to follow-up; PU, reported urgency at both enrollment and follow-up.

b Unadjusted mean difference (95% CI) between groups for continuous variables and difference in percentage points (95% CI) between groups for categorical variables.

### Differences in treatment at enrollment and changes in treatment between urgency groups

Compared to NPU patients, patients in all other fecal urgency groups (UN patients [52% vs 27%, *P* < .001], NU patients [45% vs 27%, *P* = .02], and PU patients [53% vs 27%, *P* < .001]) were more likely to change treatment between enrollment and the 6-month follow-up visit. Again, when compared to NPU patients, a higher proportion of patients in all other fecal urgency groups were on a biologic at enrollment and had any change in treatment between enrollment and the 6-month follow-up visit (UN patients [24% vs 11%, *P* = .01], NU patients [23% vs 11%, *P* = .03], and PU patients [35% vs 11%, *P* < .001]). A higher proportion of UN patients and PU received (or were receiving) 2 or more treatments at enrollment (47% and 51%, respectively) compared to NPU patients (33%; all *P* < .05) (Table 3).

**Table 3:** Association Between Ulcerative Colitis (UC) Treatment and Change in Fecal Urgency (N = 400)

	No persistent urgency (NPU)^a^ N = 175	Urgency to none (UN)^a^ N = 86	None to urgency (NU)^a^ N = 56	Persistent urgency (PU)^a^ N = 83	Chi-square *P* value (UN vs NPU)<	Chi-square *P* value (NU vs NPU)<	Chi-square *P* value (PU vs NPU)<
Treatment at enrollment, n (%)	164/175 (93.7%)	80/86 (93.0%)	52/56 (92.9%)	78/83 (94.0%)	.832	.821	.935
Biologic/JAKi only, n (%)	40/175 (22.9%)	14/86 (16.3%)	14/56 (25.0%)	15/83 (18.1)			
IST only, n (%)	4/175 (2.3%)	1/86 (1.2%)	0/56 (0.0%)	3/83 (3.6%)			
5-ASA only, n (%)	66/175 (37.7%)	29/86 (33.7%)	17/56 (30.4%)	22/83 (26.5%)			
Corticosteroid only, n (%)	2/175 (1.1%)	6/86 (7.0%)	2/56 (3.6%)	2/83 (2.4%)			
≥ 2 treatments at enrollment, n (%)	57/175 (32.6%)	40/86 (46.5%)	20/56 (35.7%)	42/86 (50.6%)	.028	.664	.005
Any change in treatment during follow-up, n (%)	48/175 (27.4%)	45/86 (52.3%)	25/56 (44.6%)	44/86 (53.0%)	< .001	.016	< .001
Biologic/JAKi only, n (%)	7/48 (14.6%)	4/45 (8.9%)	5/25 (20.0%)	8/44 (18.2%)			
IST only, n (%)	1/48 (2.1%)	3/45 (6.7%)	0/56 (0.0%)	2/83 (4.5%)			
5-ASA only, n (%)	12/48 (25.0%)	8/45 (17.8%)	5/25 (20.0%)	4/44 (9.1%)			
Corticosteroid only, n (%),	11/48 (22.9%)	10/45 (22.2%)	1/251 (44.0%)	6/44 (13.6%)			
≥ 2 changes in treatment, n (%)	25/48 (52.1%)	26/45 (57.8%)	10/25 (40.0%)	30/44 (68.2%)	.581	.327	.116
Biologic treatment at enrollment and any change in treatment, n (%)	20/175 (11.4%)	21/86 (24.4%)	13/56 (23.2%)	29/83 (34.9%)	.007	.028	< .001

JAKi, Janus kinase inhibitors; TNFI, tumor necrosis factor inhibitors; 5-ASAs, 5-aminosalicylic acids; ISTs, immunosuppressant therapies.

a NPU, no persistent urgency at enrollment or follow-up; UN, reported resolution of urgency after enrollment to follow-up; NU, reported onset of urgency after enrollment to follow-up; PU, reported urgency at both enrollment and follow-up.

**Figure 2:**
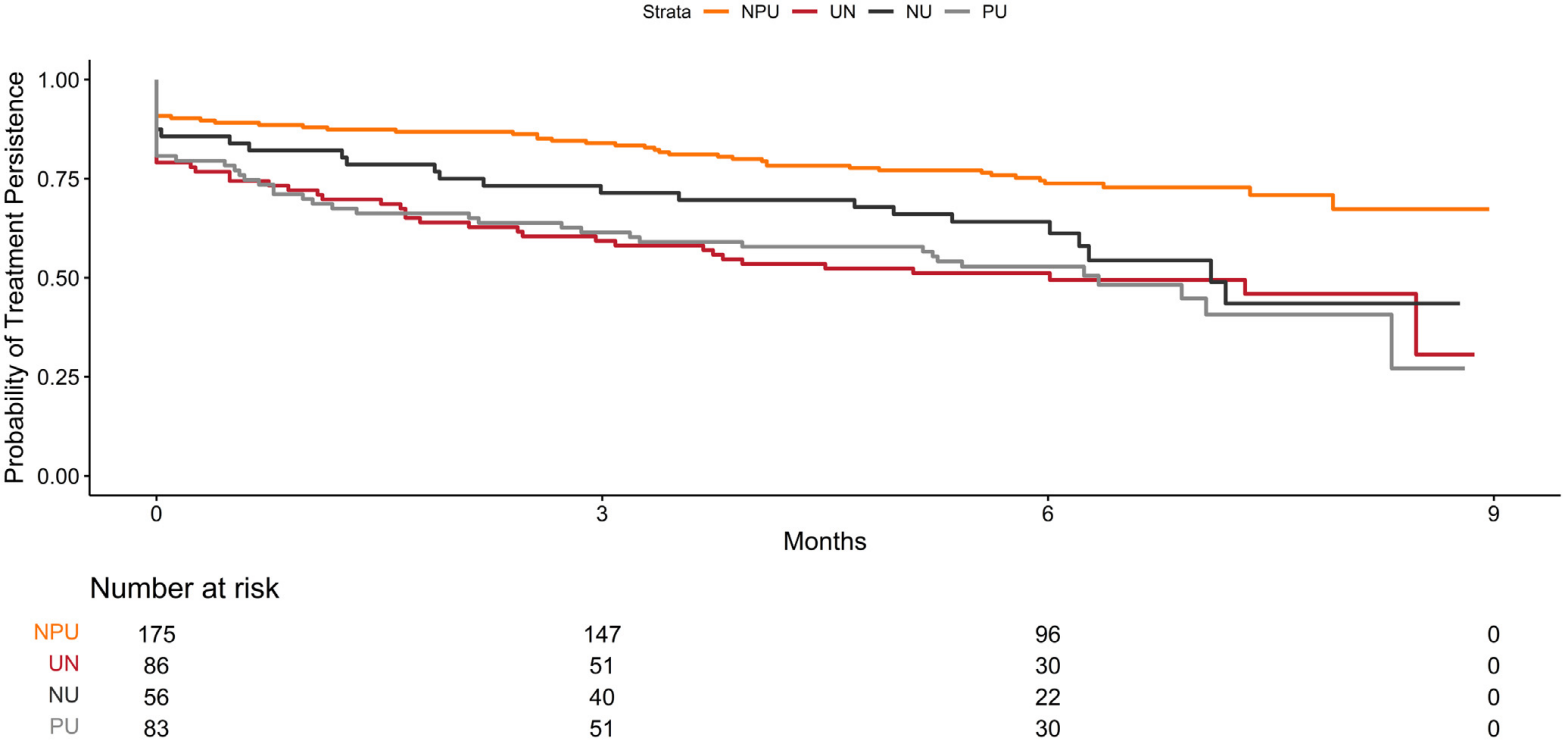
Kaplan-Meier curves: Time to first treatment change stratified by fecal urgency group.

Compared to NPU patients, the time to first treatment change was statistically different for all other urgency change groups including the PU patients (log-rank *P* value < .02 for all). However, the time to first treatment change was statistically different for NPU compared to NU patients (log-rank *P* value = .01), but not for UN patients compared to PU patients (log-rank *P* value = .93) (Figure 2). The estimated probability of persistence on treatment(s) between enrollment and follow-up was shorter for PU patients compared to NPU patients at both 3 months [61% (52%, 73%) vs 84% (79%, 90%)] and 6 months [53% (43%, 65%) vs 74% (68%, 81%)], respectively (Table 4). However, among patients in remission at enrollment, the treatments were generally similar across fecal urgency groups (data not shown).

**Table 4:** Kaplan-Meier Estimates of Treatment Change Survival at 3-Mo and 6-Mo by Urgency Group

	No persistent urgency (NPU)^a^< N = 175	Urgency to none (UN)^a^< N = 86	None to urgency (NU)^a^< N = 56	Persistent urgency (PU)^a^< N = 83	Log-rank *P* value (UN vs NPU)<	Log-rank *P* value (NU vs NPU)<	Log-rank *P* value (PU vs NPU)<	Log-rank *P* value (UN vs PU)<
Estimate of survival at 3 mo [95% CI]	0.84 [0.79, 0.9]	0.59 [0.5, 0.71]	0.71 [0.61, 0.84]	0.61 [0.52, 0.73]	*P* < .001	.012	*P* < .001	.926
Estimate of survival at 6 mo [95% CI]	0.74 [0.68, 0.81]	0.51 [0.42, 0.63]	0.64 [0.53, 0.78]	0.53 [0.43, 0.65]				

a NPU, no persistent urgency at enrollment or follow-up; UN, reported resolution of urgency after enrollment to follow-up; NU, reported onset of urgency after enrollment to follow-up; PU, reported urgency at both enrollment and follow-up; NA, Unable to estimate median and/or confidence interval limits.

## Discussion

In addition to longitudinally exploring the association between change in fecal urgency and baseline patient characteristics, to our knowledge, we are the first to further investigate the change in fecal urgency and change in treatment from enrollment to the 6-month follow-up visit. Reporting urgency at baseline was associated with a higher prevalence of comorbidities (depression, anxiety, prior infections, and diabetes) and higher burden of PROs (fatigue, pain interference, work impairment, work hours affected, and daily activities impacted). These findings indicate that fecal urgency is one factor that can diminish UC patients’ quality of life. We also found significant differences in treatment and time to treatment change between patients who experienced fecal urgency at their enrollment visit, 6-month follow-up visit, or both, compared to those without fecal urgency at any visit.

There is a lack of research at the population level that addresses potential risk factors for fecal urgency^12^^,^^23^ and few studies that evaluate different categories of fecal urgency (eg, none, changes in urgency across time, and PU).^15^ When evaluating NPU vs other change in urgency groups, we found that a higher proportion of fecal urgency occurs in females and that patients who experience fecal urgency are more than 2 times more likely to suffer from depression. Our findings align with 2 recent studies: (1) a representative sample of the US population from the National Health and Nutrition Examination Survey found an association between fecal urgency and depression and (2) Sninsky et al found that patients with an urgency level of “hurry” experienced significantly higher odds of depression (odds ratio: 3.03, 95% confidence interval, 1.82–5.15) than patients with an urgency level of “no hurry.”^15^

The inflammation associated with UC results in diarrhea, urgency, and other symptoms. In UC, the worst inflammation is typically located in the rectum. One of our more clinically relevant findings is that NU and PU patients, when compared to NPU patients, had nearly double the prevalence of proctitis, confirming the role of the rectum in fecal urgency.

Of note, we found that patients with no urgency at enrollment who later developed urgency (NU) had a higher prevalence of self-reported pain interference, moderate/severe fatigue, depression, and anxiety at enrollment than those without urgency at either visit (NPU). These NU patients who developed fecal urgency after enrollment worked less and had more corticosteroid experience than NPU patients. We found that nearly 45% of NU patients changed treatments which we think may be due to worsening disease. Yet, we did find some differences in PROs between the groups. Determining potential reasons for these findings is restrained by our relatively limited sample size and duration of this study. We need more patients with a longer follow-up time to see if the NU patients’ symptoms resolve following treatment change. However, in cases such as this, providers may be able to identify the patients who are more likely to relapse using PROs at routine visits and change their intensity of monitoring and/or treatment course, which is strength of this study. As the CorEvitas registry grows, we will conduct more extensive studies to answer these questions as we will not be constrained by limited sample size.

The presence of urgency may indicate underlying inflammation that is not being adequately controlled. Additional diagnostic testing, such as biomarkers or endoscopy, are needed to distinguish whether inflammation vs other etiologies (functional overlap, structural damage to the colon) are causing the symptoms. Our study found that patients with fecal urgency at enrollment or the 6-month follow-up visit (UN and PU) had an earlier treatment change than NPU patients. This association is intuitive, as providers likely changed treatment to address the urgency symptoms and underlying inflammation causing the symptoms. Furthermore, it highlights that urgency may drive the need for multiple treatment changes. However, our study not determines if urgency was the driving factor of treatment change or if other related symptoms that often co-occur (eg, rectal bleeding, stool frequency) were either meaningfully contributing or perhaps the primary drivers of treatment change. While not possible with the data available in this study, delineating the temporal sequence of symptoms associated with active inflammation help elucidate which symptoms are most strongly associated with active inflammation and the need to consider treatment changes.

We recognize that our study has some limitations. Although the SCCAI is considered a reasonable measure of real-world disease activity^24^ and our stratification by disease activity partially accounts for symptoms from other components of the SCCAI, we acknowledge this is a limitation compared to having objective measures of disease activity. Moreover, we cannot eliminate the possibility that the differences we found were not due to co-occurring symptoms. We further note that comparisons were made to highlight the descriptive differences in patient characteristics at enrollment by the change in urgency status and should not be construed as differences between responses to treatment. As in all geographically restricted registries, the patient population might not be generalizable to UC patients outside the United States. Finally, the 67 participating sites at the time of this study included both academic (n = 10) and private practice clinics (n = 57), and these participating physicians in the CorEvitas IBD Registry may not represent clinical practice patterns at large. Despite these limitations, this study provides important insights. Our study involved real-world systematically collected data that provide a more realistic view of the burden of disease and unmet needs in UC patients. Our patient population is more reflective of the general US UC population than those in UC clinical trials.

## Conclusion

Fecal urgency is a UC symptom with great clinical impact. Studies have consistently shown its association with psychological disorders, decreased quality of life, and a strong predictor of fecal incontinence. Our study cannot determine which symptoms or constellation of symptoms is the main driver in changing treatment. However, our results suggest that urgency may be a key symptom and should be assessed at each clinical visit. Fecal urgency at any stage in a patient’s disease course may be a sign of inadequate therapy and often indicates the need for modifying treatment. Patients with fecal urgency are more likely to change treatment and to do so earlier. Therefore, identification of fecal urgency warrants further assessment for inflammation in the colon or other causes of urgency and conceivably more intense monitoring to identify recurrent colitis symptoms earlier in the disease course.
